# The feasibility of incorporating structured therapeutic consultations with real patients into the clinical clerkship internal medicine

**DOI:** 10.1007/s00210-012-0775-6

**Published:** 2012-08-17

**Authors:** R. J. van Unen, J. Tichelaar, A. J. Schneider, E. C. T. Geijteman, P. W. B. Nanayakkara, A. Thijs, M. C. Richir, Th. P. G. M. de Vries

**Affiliations:** 1 Department of Clinical Pharmacology and Pharmacy, VU University Medical Center, De Boelelaan 1117, 1081 HV Amsterdam, The Netherlands; 2 Department of Internal Medicine, VU University Medical Center, Amsterdam, The Netherlands

**Keywords:** Clerkship, Consultations, Medical students, Prescribing, Pharmacotherapy

## Abstract

This study aims to determine the feasibility of incorporating structured therapeutic consultations (TCs) into the clinical clerkship internal medicine. TCs were considered feasible if students were able to draw up a therapeutic plan and carry out a TC, and if students and their supervisors considered TCs workable and useful. From March 2008 to October 2009, medical students carried out a “diagnostic” and subsequent “therapeutic” consultation with the same patient during their clinical clerkship internal medicine at the VU University Medical Center. After the diagnosis was established, the student had to formulate a therapeutic plan and then carry out a TC with the patient, supervised by a clinician. The supervisor assessed the therapeutic plan and how the student conducted the TC. Both the student and the supervisor received a questionnaire about the workability and usefulness of the TC. On average, students' performance in drawing up a therapeutic plan was awarded a score of 4.4 on a five-point scale, and the TC performance of 96 % of the students was considered amply sufficient or better. Eighty-three percent of the supervisors agreed or strongly agreed with the statement that the TC is a worthwhile addition to the clerkship, and 67 % of the students indicated that they would like to perform more TCs. This study shows that incorporating a structured TC with a real patient into the clinical clerkship internal medicine is both feasible and worthwhile. This may be an important step to improving the prescribing skills and attitudes of junior doctors and residents and to reducing their prescribing errors after graduation.

## Introduction

The main objective of medical curricula is to provide graduates with diagnostic and therapeutic skills and competencies. Broadly speaking, medical curricula are divided into two phases, a preclinical phase followed by a clinical (clerkship) phase. During the preclinical phase, emphasis is usually on the acquisition of theoretical knowledge and skills in the diagnosis and treatment of diseases, while during the clinical phase, this knowledge and these skills are put into practice. However, especially during the clinical phase, attention tends to be on the acquisition of diagnostic, rather than therapeutic, skills. In this phase of their training, medical students perform consultations with new patients in the outpatient departments, take a detailed history, perform a physical examination, and determine the differential diagnosis and formulate a diagnostic plan. However, they seldom work out a therapeutic plan during the initial consultation and are rarely involved in the next step, the therapeutic consultation (TC), during which the diagnosis is discussed with the patient, and a therapeutic strategy is chosen and started. Students also rarely carry out the follow-up consultations in which the effect of therapy is monitored. To our knowledge, this situation applies to most Dutch medical schools and probably also to most medical schools abroad.

It is, therefore, not surprising that many junior doctors feel that they are not prepared for their responsible roll as house officers regarding therapeutic treatments (Tobaiqy et al. 2007; Heaton et al. 2008; Prince et al. 2004; Han and Maxwell 2006), and that they make many prescribing errors (Dean et al. 2002b; Lesar et al. 1990). Also, there is no clear reason why more time cannot be devoted to therapeutic aspects during the clinical clerkship. Often-heard reasons for not doing this are: that students will learn about therapy later during their registrar period, and that they are not yet ready to treat real patients. We doubt this, and this is why we developed a pharmacotherapy training assignment at the outpatient department of internal medicine at the VU University Medical Center, Amsterdam, The Netherlands, during which students carry out a diagnostic and a therapeutic consultation with the same patient. The aim of this study was to determine the feasibility of incorporating structured TCs into the clinical clerkship internal medicine. TCs were considered feasible if students were able to draw up a therapeutic plan and carry out a TC, and if students and supervisors considered the TC workable and useful.

## Method

### Population

From March 2008 to October 2009, all medical students, who performed their clinical clerkship in internal medicine at the VU University Medical Center, were included in the study. Prior to this clerkship, all included students had completed a preclinical context-learning pharmacotherapy training program during the second to fourth years of their study, as described previously (Vollebregt et al. 2006). In short, this program consisted of weekly role-playing sessions in the form of a consultation during which “student” doctors perform therapeutic consultations with student patients. These consultations are observed by and discussed with student assessors. After the consultations and under the supervision of a clinical pharmacologist, the students discuss the various (drug) treatment options and how they performed as doctors.

### Pharmacotherapy training assignment

The pharmacotherapy training assignment consisted of carrying out a diagnostic and a therapeutic consultation with the same patient who visited the outpatient clinic of internal medicine for the first time (see Box 1). After the student had performed the diagnostic consultation, an appointment was made for the TC. A few days before the TC, the student and the supervising clinician determined the diagnosis based on the results of diagnostic tests, and the student formulated a therapeutic plan based on the WHO six-step plan (Vries de et al. 1995). Since this was patients' first visit to the outpatient clinic, there were no existing therapeutic plans available. Therefore, all students had to formulate their own therapeutic plan. The therapeutic plan involved the written completion of the following six steps: step 1, define the indication for the treatment; step 2, specify the therapeutic objective; step 3, specify the standard treatment for the diagnosis; step 4, choose a preliminary (drug) treatment, taking all relevant patient characteristics into account; step 5, “write a prescription” in the case of drug treatment and determine what information should be given to the patient; and step 6, determine what should be measured and when, in order to monitor the progress of treatment. The therapeutic plan was discussed with and evaluated by the supervisor. Because it is known that clinical supervisors tend to overestimate performance scores and hardly fail a student during clerkships (Williams et al. 2003), step 4 (choose treatment) and step 5 (write prescription and determine patient information) were also assessed by an independent assessor not involved in the supervision of these students. Subsequently, the student carried out the TC and, together with the patient, determined the definite therapeutic plan again supervised by the clinician. The supervisor then evaluated (both orally and in writing) student's performance during the TC. Lastly, student and supervisor completed a questionnaire on the workability and usefulness of the TC (Table 2).

**Box 1** The pharmacotherapy training assignment (diagnostic + therapeutic consultation) during the clinical clerkship internal medicine
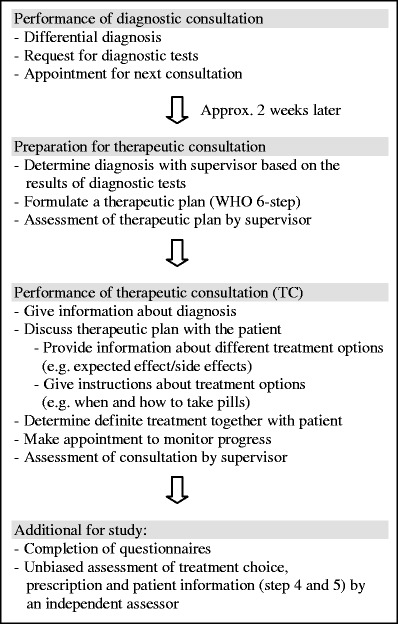


### Scoring and analysis

Performance on each step of the therapeutic plan was scored 1 to 5 (1 = lowest attainable score and 5 = maximum attainable score), as was TC performance (1 = inadequate; 2 = doubtful; 3 = sufficient; 4 = amply sufficient; 5 = good). The students were asked to indicate how long they took to prepare for the TC (1 = 1–3 h; 2 = 4–6 h; 3 = 7–10 h; 4 ≥ 10 h). In the questionnaire evaluating the workability and usefulness of the TC, agreement with a number of statements was scored on a five-point scale (1 = strongly disagree; 2 = disagree; 3 = neutral; 4 = agree; 5 = strongly agree).

The data were analyzed using SPSS 15.0 (SPSS, Chicago, IL). Differences were analyzed using a Wilcoxon signed rank test. A *P* value of <0.05 was considered statistically significant.

## Results

From March 2008 to October 2009, 50 of 86 eligible students completed the diagnostic and therapeutic consultations with the same patient. Thirty-six (42 %) students did not carry out the TC. Twelve clinical specialists supervised and evaluated the students. An overview of the diagnoses that were subject of the therapeutic plans is given in Table 1. Students' mean scores for drawing up a therapeutic plan and carrying out a TC and the preparation time are shown in Table 2. The supervisors gave the overall therapeutic plan (steps 1 to 5) a score of 4.4, with treatment choice (step 4) being scored 4.3, and prescription and patient information (step 5) being scored 4.4. The independent assessor gave step 4 a score of 3.7 and step 5 a score of 4.4. TC performance was assessed as doubtful in 4 % of students, amply sufficient in 48 % of students, and good in 48 % of students. Seventy-one percent of the students needed 1–3 h to prepare for the TC; 24 %, 4–6 h, and 5 %, 7–10 h.

**Table 1:** Overview of the diagnoses for which a therapeutic plan was made during the training assignment

Diagnosis	Number of therapeutic plans
Gastrointestinal diseases	10
Hypertension	9
Hypo-/hyperthyroidism	9
Infectious diseases	5
Iron-deficiency anemia	5
Diabetes mellitus	4
Osteoporosis	3
Hypercholesterolemia	2
Atrial fibrillation	1
Depression	1
Diabetes insipidus	1
Total	50

**Table 2:** Scores for the therapeutic plan (max = 5; *n* = 50), for the therapeutic consultation (*n* = 50), and for the time taken to prepare for the consultation (*n* = 45)

	Average score (95 % CI)	Score independent	Percent
	Supervisor	Assessor	
Preparation therapeutic plan			
Step 1: Define indication	4.4 (4.2–4.6)		
Step 2: Specify therapeutic objective	4.3 (4.1–4.5)		
Step 3: Specify standard treatment	4.3 (4.1–4.5)		
Step 4: Choose a (drug) treatment	4.3 (4.1–4.5)	3.7 (3.5–3.9)*	
Step 5: Write prescription and determine information	4.4 (4.2–4.6)	4.4 (4.2–4.6)	
Step 6: Determine monitoring parameters	4.4 (4.2–4.6)		
Performance therapeutic consultation			
In adequate			0
Doubtful			4
Sufficient			0
Amply sufficient			48
Good			48
Preparation time students			
1–3 h			71
4–6 h			24
7–10 h			5
>10 h			0

*p < 0.001

Forty-five (90 %) students, who carried out both consultations, completed the questionnaire, as did all 12 supervisors (Table 3). Regarding the workability of this approach, 84 % of the students agreed or strongly agreed that they had enough preparation time, and 71 % agreed or strongly agreed that they could easily carry out a TC in combination with their other clinical activities. Seventeen percent of the supervisors thought that the TC approach involved too much work, whereas 66 % were neutral about the work involved, and 17 % thought that it did not cost them too much time. With respect to the usefulness of this approach, 93 % of the students agreed or strongly agreed that they liked being able to consult the patient for a second time, and 67 % indicated that they would like to perform more TCs. Eighty-three percent of the supervisors agreed or strongly agreed that the TC is a worthwhile addition to the clerkship internal medicine.

**Table 3:** Supervisor (*n* = 12) and student (*n* = 45) ratings of the workability and usefulness of the therapeutic consultation

	Strongly disagree (%)	Disagree (%)	Neutral (%)	Agree (%)	Strongly agre (%)
Workability					
Supervisors					
Supervision of the consultation involved too much work	0	17	66	17	0
The instructions were clear	0	0	8	50	42
My role was clear	0	0	17	33	50
Students					
I had enough time to prepare for the consultation	0	4	16	50	34
The therapeutic consultation can easily be combined with other activities	4	7	18	51	20
The instructions were clear	0	2	14	62	22
Usefulness					
Supervisors					
Carrying out a therapeutic consultation is a good addition to the clerkship	0	0	17	47	36
Students					
I liked seeing a patient for a second time	0	0	7	36	57
I was satisfied with clinician's supervision	0	4	16	39	41
I would like to perform other therapeutic consultations	2	7	24	31	36

## Discussion

This study shows that it is feasible to incorporate a structured TC with a real patient into the clinical clerkship internal medicine. On average, students' performance in drawing up a therapeutic plan and carrying out a TC was more than sufficient. Both clinicians and students considered the extra work and effort involved worthwhile, and students appreciated the opportunity to carry out a TC.

The uniformly high scores (>4) for the therapeutic plan are remarkable, but are consistent with an earlier report of scores for therapeutic performance of about 80 % of the maximum attainable score (Richir et al. 2008). We are aware that clinical supervisors tend to overestimate performance scores and hardly fail a student during clerkships (Williams et al. 2003), so the scores must be interpreted carefully. The independent assessor scored the treatment choice of students (step 4) significantly (*p* < 0.001) lower than did the supervisors, although the score was still sufficient. This difference might be due to the context in which the assessments took place. The supervisors knew the patients from the diagnostic consultation and had access to patients' medical records, which might have influenced their evaluation of students' choice of treatment. Despite this difference, the results show that supervising clinicians agree that trained medical students are sufficiently competent to carry out supervised TCs with real patients. Regarding the appreciation for the TC, earlier studies have also shown that students appreciate supervised interaction with real patients (Murray et al. 2001; Griffith et al. 2009). Furthermore, TCs with real patients constitute an optimal form of context learning, which is an effective learning method (Vollebregt et al. 2006; Richir et al. 2008; Charlin et al. 2007; Mann 2002; Schmidt and Rikers 2007; Chastonay et al. 1996). According to Coles (1998), a criterion for context learning is repetition. In this study, the students performed only one consultation at the start of their clerkship. Thus for optimal learning, students should have the opportunity to carry out other TCs during subsequent clerkships. Moreover, the preparation time might become shorter if students perform more TCs. At the moment, 29 % of the students needed 4 h or longer to prepare for the TC. This might be because they were confronted with diseases that had not been dealt with during the preclinical phase of the medical curriculum; however, subsequent analysis revealed that the preparation time was similar for clinical problems that had been covered during the preclinical pharmacotherapy program (data not shown). This so-called transfer effect has been described earlier (Richir et al. 2008). Since it was the first time that students carried out a TC with a real patient, they probably did not know what to expect and wanted to leave nothing to chance.

Another benefit of performing TCs during outpatient clinics is that students have to carry out the TC within the time available and thus learn to cope with time pressure. Since time pressure is a recognized cause of prescribing errors (Dean et al. 2002a; Nichols et al. 2008), early exposure to time pressure might prevent medication errors later during the junior doctor period.

In spite of the optimal context and the fact that students appreciated the consultations, 36 (42 %) students did not manage to see a patient a second time for a TC. Possible reasons for this are that diagnostic test results were not back before students rotated to another clerkship, or the second appointment could not be made within this 4-week period. These results suggest that other forms of TCs are needed to enable all students to carry out a TC. An alternative is for students to carry out TCs with patients they have not seen in a diagnostic consultation or for students to carry out follow-up consultations, during which therapy is monitored.

Recently, Celebi et al. (2010) showed that medical students do not acquire adequate prescribing skills by merely watching other doctors prescribe during clerkships. Instead, this might encourage undesired copying behavior (Tichelaar et al. 2010). So, despite the above-mentioned limitations, allowing students to carry out TCs with real patients in the clinical phase of the medical curriculum is a valuable component of clinical clerkships. According to our knowledge, this is the first study that investigated if it is feasible that students carry out a TC with real patients during the clinical phase of the medical curriculum. Future studies should focus on the effect of this pharmacotherapy training assignment on the confidence of junior doctors and, more importantly, the prevention of prescribing errors.
